# Driving force of biomolecular liquid–liquid phase separation probed by nuclear magnetic resonance spectroscopy

**DOI:** 10.52601/bpr.2022.210034

**Published:** 2022-04-30

**Authors:** Hanyu Zhang, Weiwei Fan, Gilbert Nshogoza, Yaqian Liu, Jia Gao, Jihui Wu, Yunyu Shi, Xiaoming Tu, Jiahai Zhang, Ke Ruan

**Affiliations:** 1 Ministry of Education Key Laboratory for Membraneless Organelles & Cellular Dynamics, Hefei National Laboratory for Physical Sciences at the Microscale, School of Life Sciences, Division of Life Sciences and Medicine, University of Science and Technology of China, Hefei 230027, Anhui, China; 2 Department of Applied Chemistry, College of Science and Technology, University of Rwanda, Kigali, Rwanda

**Keywords:** Membraneless organelles, Liquid–liquid phase separation, NMR spectroscopy, Chemical shift perturbation, Paramagnetic relaxation enhancement

## Abstract

The assembly of biomolecular condensates is driven by liquid–liquid phase separation. To understand the structure and functions of these condensates, it is essential to characterize the underlying driving forces, *e*.*g*., protein–protein and protein–RNA interactions. As both structured and low-complexity domains are involved in the phase separation process, NMR is probably the only technique that can be used to depict the binding topology and interaction modes for the structured and nonstructured domains simultaneously. Atomic-resolution analysis for the intramolecular and intermolecular interactions between any pair of components sheds light on the mechanism for phase separation and biomolecular condensate assembly and disassembly. Herein, we describe the procedures used for the most extensively employed NMR techniques to characterize key interactions for biomolecular phase separation.

## INTRODUCTION

In eukaryotic cells, compartments provide spatiotemporal regulation over specific functions (Lin* et al.* 2015). These compartments are either membrane-bound organelles, *e*.*g*., lysosomes, synaptic vesicles, or membraneless organelles (MLOs), such as nuclear speckles, stress granules, and processing bodies. Many of these MLOs were identified decades ago, but the assembly, disassembly, material exchange of MLOs, and the contribution of their physicochemical properties to biological functions remain elusive. Pioneering work has revealed that P granules in *C. elegans* collide and coalesce like oil in water, as the liquid-liquid phase separation (LLPS) process is a bread-and-butter concept in chemistry and physics. Phase separation is driven by multiple weak and multivalent interactions to concentrate certain molecules and exclude others; thus, compartmentalization is realized to fulfill specific biological functions in the crowded chaos of the cell (Vernon* et al.* 2018). The formation of MLOs driven by LLPS facilitates our understanding of their diverse functions, *e*.*g*., stress response, signal transduction and gene expression (Courchaine* et al.* 2016; Ryan* et al.* 2018). The dysregulation of MOLs is, therefore, related to a variety of diseases, *e*.*g*., amyotrophic lateral sclerosis (Conicella* et al.* 2016; Kim* et al.* 2013), Alzheimer’s disease (Ambadipudi* et al.* 2019), and chronic traumatic encephalopathy (McKee* et al.* 2010).

To understand the organization of MLOs, it is necessary to reconstitute the components *in vitro* to describe the driving forces for LLPS. As MLOs are in dynamic equilibrium between assembly and disassembly harboring hundreds of RNAs and proteins (Conicella* et al.* 2020; Jonas and Izaurralde 2013; Murthy* et al.* 2019; Nott* et al.* 2015; Ribbeck and Gorlich 2002; Teixeira* et al.* 2005; Tsai* et al.* 2016), only the core nucleating components are practically singled out to probe the correlation between intermolecular interactions and *in vitro* phase separation diagrams. A variety of techniques, as described previously for the same issue, have been used to investigate the phase diagram, structure, dynamics and function of these ribonucleoprotein droplets (Shin* et al.* 2018). NMR spectroscopy has gained increasing popularity in this field due to its capacity to detect multiple weak interactions among structured and low complexity domains (LCDs) (Luna* et al.* 2014; Musielak* et al.* 2020; Vaynberg and Qin 2006). Here, we describe the procedures for these NMR experiments, *e*.*g*., chemical shift perturbations (CSPs) and paramagnetic relaxation enhancement (PRE), which have been extensively used in phase separation studies. This structural information guides the rational design of loss-of-function mutants for LLPS and granule formation studies.

## NMR SAMPLE PREPARATION

**Figure 1:**
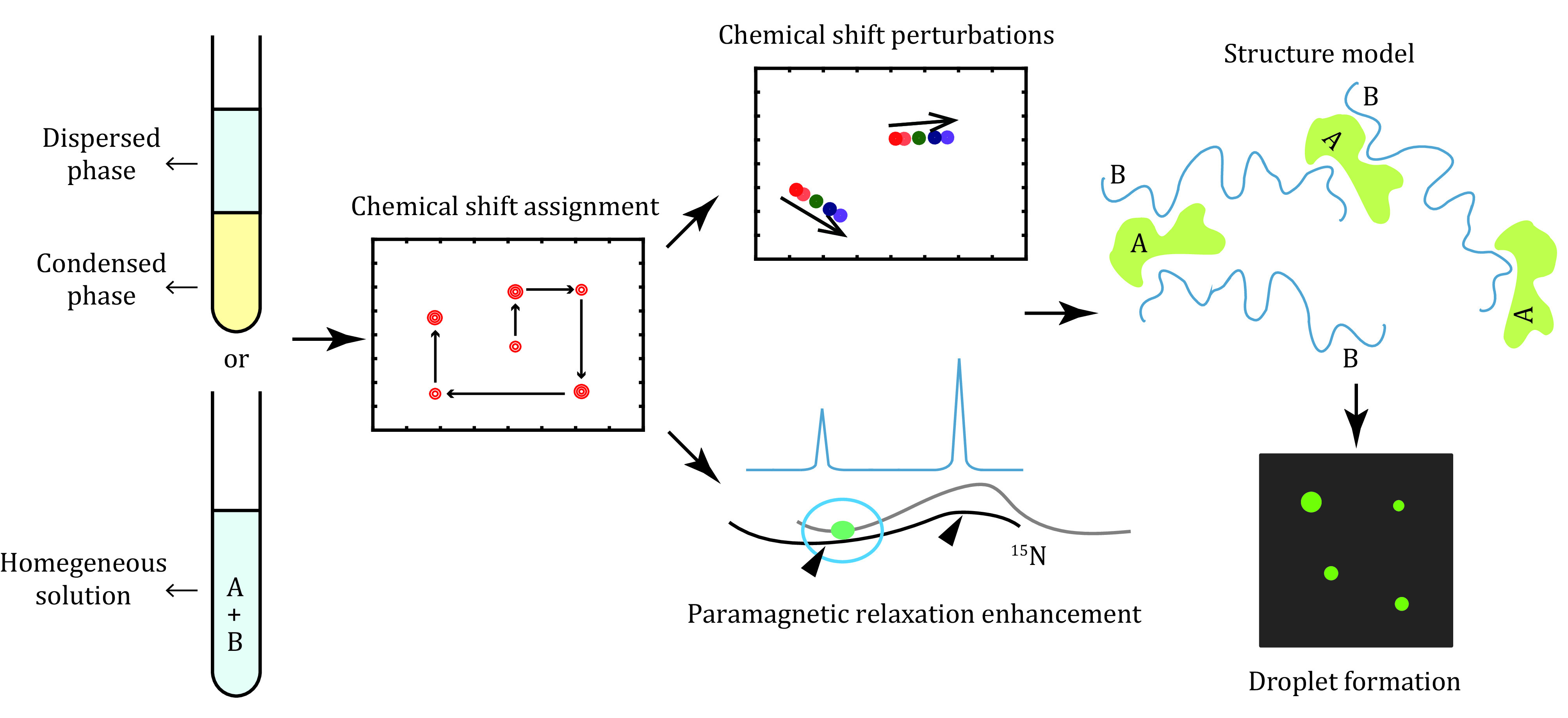
Scheme for the characterization of intermolecular interactions key to liquid-liquid phase separation using NMR spectroscopy

The recombinant protein was prepared individually and stored in a buffer to stabilize the protein in a homogenous state. There were two ways to probe the intermolecular interactions key to LLPS using NMR spectroscopy (Fig. 1). One way is to dilute the protein to LLPS buffer or mix it with its partners to prepare a biphase sample, which is then centrifuged to separate the dilute phase from the condensed phase. Although NMR spectra can provide structural and dynamic information for this phase-separated protein, this technique suffers from low sensitivity due to the high viscosity of condensates relative to the soluble and dispersed protein. An alternative way is to depict the key intermolecular interactions under homogeneous conditions using NMR spectroscopy. The interactions key to LLPS are then cross validated by mutagenesis, posttranslational modification or small molecule inhibitors in the optics-based droplet formation assay. Preparation of these protein samples with different isotope labeling or posttranslational modifications is described below.

### Nonlabeled protein expression and purification

DNA encoding the full-length or selected domain of the protein of interest was amplified and inserted into a plasmid containing the His, SUMO, GST or MBP tag in the N- or C-terminus of the target. A tobacco etch virus (TEV) cleavage site or a thrombin protease site was encoded between the target gene and the aforementioned tag. We selected a tag with a molecular weight that significantly deviated from that of the target protein, thus favoring protein purification after cleavage.

*Escherichia coli* cells were cultured in LB medium at 37 °C until OD_600nm_ reached a value of 0.8–1.2 and induced by 0.1–1.0 mmol/L isopropyl-β-D-thiogalactopyranoside (IPTG), usually at 16 °C for 24 h or 37 °C for 6 h. The bacterial pellet was resuspended and lysed by sonication on ice or a high-pressure homogenizer.

The supernatant was purified on beads and then treated with TEV or thrombin, based on the type of fused tag used, overnight at 16 °C. It was then purified using size exclusion chromatography columns, *e*.*g*., Superdex 75 or 200, and ion-exchange columns when a higher purity was desired.

Finally, the purified protein was concentrated and stored in an optimized buffer at –80 °C to achieve long-term stability. A typical buffer contained 150 mmol/L NaCl, 2 mmol/L DTT, and 1 mmol/L EDTA. A high salt concentration was found to be usually favored, and the reducing agent concentration was usually five to ten times excess relative to the equivalent amount of cysteine residues. For example, if a protein containing three cysteine residues was to be concentrated at 0.1 mmol/L, the DTT or TCEP concentration was set at 1.5 to 3.0 mmol/L. Sodium azide (NaN_3_, 0.02% weight) was added for a long-term experiment.

### Isotope labeled protein

The same reconstructed plasmids were transformed into *Escherichia coli* cells, which were cultured in LB medium until an OD_600nm_ value of 0.8–1.2 was reached and then transferred to a minimal medium supplemented with ^15^NH_4_Cl for ^15^N labeling only or ^15^NH_4_Cl and ^13^C glucose for uniform [^15^N, ^13^C] labeling. The remaining procedures were the same as those used for the nonlabeled protein samples. The ^15^N-labeled protein was designated for chemical shift perturbations (CSPs) and paramagnetic relaxation enhancement (PRE) experiments, while the [^15^N, ^13^C]-labeled sample was designated for backbone chemical shift assignment. The latter sample was used in the intermolecular interaction studies as well, where three-dimensional experiments were applied to lift severe signal degeneration for LCDs.

### Paramagnetic labeled protein

This sample was specifically used for PRE measurements. The target protein was mutated to introduce only one Cys in the designated site. If the target protein contains a limited number of Cys residues in its native sequence, these residues should be mutated to Ala or Ser first. Mutagenesis should proceed with caution to induce as little interference as possible with the native structures and intermolecular interactions. Therefore, three or more mutants, each containing one Cys at a different site, should be prepared following the same procedure as that used for the nonlabeled or ^15^N-labeled protein. The mutant was then diluted to approximately 0.1 mmol/L with a 5-fold molar excess of a reducing agent such as DTT or TCEP. After incubation at room temperature for 2 h, the reducing agent was removed by a gel filtration column (Sephadex G-25 or equivalent) or concentrated and then diluted three times. MTSL at an 8-fold excess was added to the protein solution and incubated for 8 h at room temperature or overnight at 4 °C. The excess MTSL was then removed through dialysis or in the same way as for removing the reducing agent.

### Preparation of the posttranslational modification sample

Cotransformation was used to simultaneously express the target protein and its enzyme, *e*.*g*., PRMT1. The two plasmids were constructed with different antibiotic resistances to ensure the success of cotransformation in the presence of the two antibiotics. Additionally, the expression and purification of modified proteins were the same as that described in the section "Nonlabeled protein expression and purification". The posttranslational modification of the protein was confirmed by mass spectrometry.

## NMR CHEMICAL SHIFT ASSIGNMENT

To depict the interactions between a pair of biomolecules at atomic resolution, the first step is to assign the chemical shift to a specific atom. It is worth noting that the chemical shift assignment can be directly transferred from that of the target protein or its homolog with high sequence identity from the Biological Magnetic Resonance Bank (https://bmrb.io). For the case of no assignment released, the following sequential assignment was carried out to connect every single chemical shift observed on a set of 2D, 3D and even 4D NMR spectra, which provide intra- and interresidue backbone and side-chain correlations. This crucial step is essential to initiate any structural or dynamic study by NMR. The assignment strategies can vary for proteins with different molecular weights. In general, homonuclear ^1^H-^1^H 2D NMR experiments are sufficient for proteins with molecular weights of less than 10 kDa. Multidimensional heteronuclear NMR spectra were acquired for large proteins with a molecular weight ranging from 10 to 40 kDa; in such cases, the proteins were isotope labeled, *e*.*g*., uniformly [^15^N, ^13^C]-labeled, and perdeuterated if necessary. Selective labeling of ILV methyl groups has empowered NMR to probe supermolecular complexes with molecular weights of over 1 MDa. Herein, we describe the most extensively used experiments for assigning the backbone chemical shifts for a structured domain or an LCD.

### Peptides and small proteins

Peptides and small proteins with molecular weights of less than 10 kDa were assigned to a certain extent depending on the samples’ molecular weight, folding state, or spectrometer resolution using ^1^H–^1^H correlations (COSY) (Aue* et al.* 1976; Nagayama* et al.* 1980), total correlation spectroscopy (TOCSY) (Piotto* et al.* 1992; Sklenar* et al.* 1993) and NOESY (Jeener* et al.* 1979; Wagner and Berger 1996). COSY spectra are used to build the ^3^J_HH_ correlations, while TOCSY spectra offer the correlation among the amide, H^α^, and side-chain protons. NOESY spectra provide spatial correlation for any pair of protons within 5 Å. This involves three main steps:

(1) Identification of amino acid types from their characteristic spin-system network using COSY and TOCSY spectra.

(2) Ascribing these networks to the corresponding amide protons.

(3) Validation of the sequential connectivity using NOESY spectra (Wagner and Berger 1996).

The NMR spectra were processed by NMRpipe and further analyzed by Sparky, NMRView, or CARA.

### Folded domains

**Figure 2:**
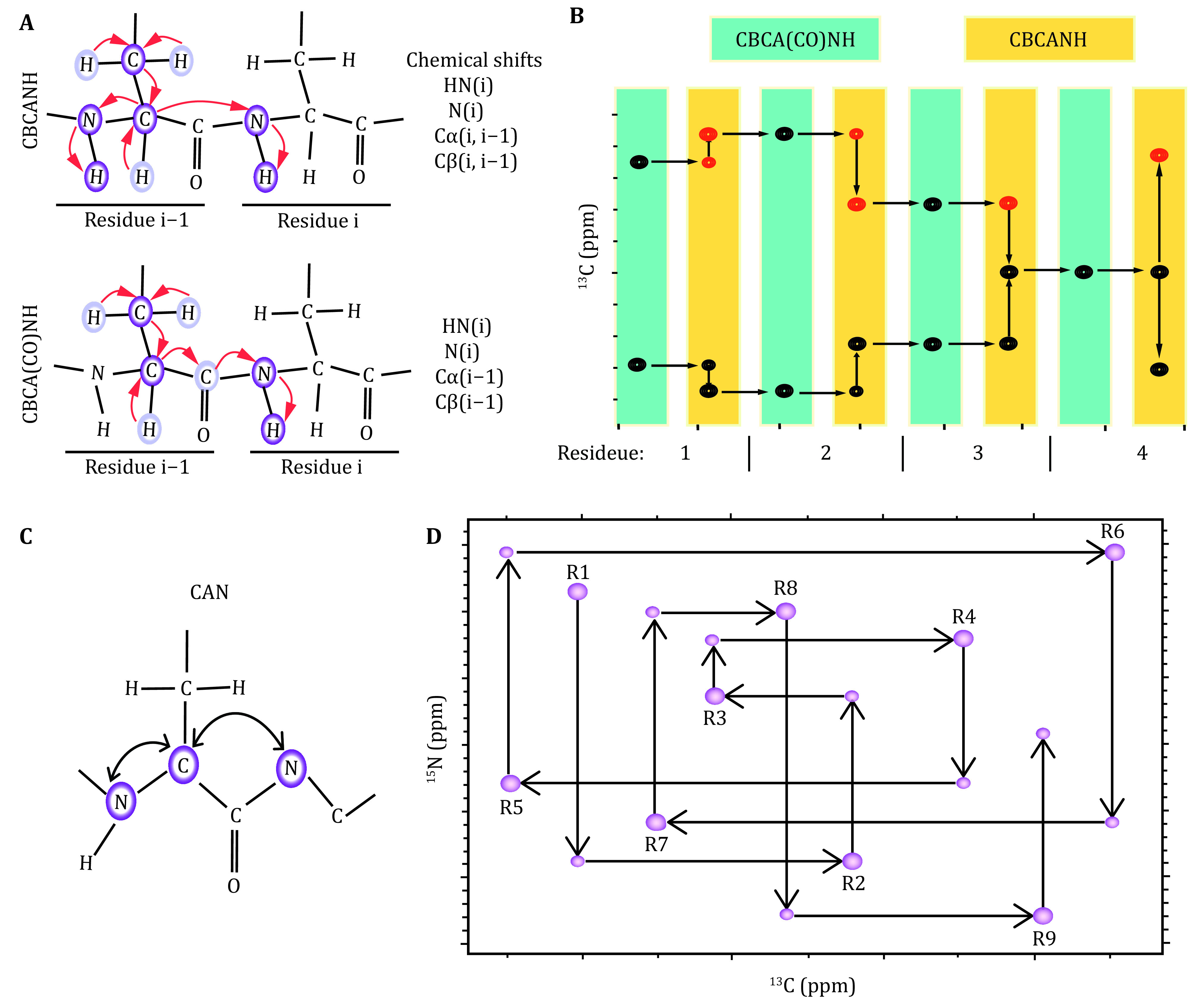
Illustration of sequential assignment of protein backbone chemical shifts. **A** Inter- and intraresidue chemical shift connectivity in the CBCA(CO)NH and CBCANH pulse sequences. **B** Sequential assignment using CBCA(CO)NH and CBCANH spectra to “walk” through the backbone chemical shifts in a stairwise manner. **C** Correlations observed in a 2D CAN –HSQC-IPAP experiment using [^13^C, ^15^N]-labeled samples. **D** Sequential assignment using CAN experiments for low complexity domains

A set of 3D heteronuclear NMR spectra, *e*.*g*., HNCA and HN(CO)CA (Grzesiek and Bax 1992b; Ikura* et al.* 1990a, b; Yamazaki* et al.* 1994a, b), HNCO and HN(CA)CO (Ikura* et al.* 1990b;Kay* et al.* 1994; Matsuo* et al.* 1996; Muhandiram and Kay 1994; Yamazaki* et al.* 1994a), and CBCANH and CBCA(CO)NH (Grzesiek and Bax 1992a, c; Ikura* et al.* 1990b; Muhandiram and Kay 1994), HA(CA)NH, HA(CACO)NH, were acquired for a uniformly [^15^N,^13^C]-labeled protein. The chemical shifts and their connectivity were retrieved from the respective spectra. The interresidue connectivity was built based on the ^2^J correlation between C^α^ and amide N. All these experiments were designed to “walk” through the protein’s backbone. For example, the CBCA(CO)NH experiment correlates the C^α^ and C^β^ chemical shifts for residue *i* – 1 with the ^1^H and ^15^N chemical shifts for residue *i*. Accordingly, the HNCACB or CBCANH experiment builds the inter- and intraresidue correlation for C^α^ and C^β^ chemical shifts for residue *i* and *i* – 1 with the ^1^H and ^15^N chemical shifts for residue *i* (Fig. 2A).

The combination of these experiments, therefore, enables us to “walk” through the protein backbone atoms. Due to the sensitivity and signal degeneracy of the CBCANH and CBCA(CO)NH spectra, supplementary spectra were all acquired to achieve a near complete assignment of all backbone chemical shifts. The characteristic chemical shifts for specific residues are listed below (http://www.bmrb.wisc.edu):

(1) Ala, 15 ppm < C^β^ < 20 ppm;

(2) Gly, no Cβ, C^α^ ~ 45 ppm;

(3) Arg, Gln, Glu, His, Lys, Met, Val, Trp, 20 ppm < C^β^ < 40 ppm;

(4) Asp, Asn, Ile, Leu, Phe and Tyr, 38 ppm < C^β^ < 52 ppm;

(5) Ser and Thr, 65 ppm < C^β^ < 75 ppm;

(6) Some Val residues, C^α^ > 64 ppm and 25 ppm < C^β^ < 36 ppm;

(7) Some Ile residues, C^α^ > 64 ppm and 36 ppm < C^β^ < 52 ppm.

A variety of other experiments are available for side-chain chemical shift assignment, such as, H(CCO)NH, (H)C(CO)NH, and HCCH–TOCSY (Grzesiek and Bax 1993; Kay* et al.* 1993; Montelione* et al.* 1992; Schwalbe* et al.* 1993), which helps identify the residue type.

### Low complexity domains

The multivalent weak interactions among the LCDs are one of the key driving forces for LLPS (Borcherds* et al.* 2021). The challenge is that LCDs are intrinsically flexible; thus, they can interconvert between various conformational states (Jensen* et al.* 2014). Another challenge is the signal degeneration of amide protons due to the low sequence complexity and disordered structures; in such cases, ^13^C or ^15^N direct detection experiments can be performed (Bermel* et al.* 2006a; Eletsky* et al.* 2003; Pervushin and Eletsky 2003; Serber* et al.* 2000, 2001; Takeuchi* et al.* 2008). The spatial arrangement of the ^13^C or ^15^N coil was optimized to enhance the sensitivity of these two nuclei (Kovacs* et al.* 2005).

The 2D NCA experiment correlates the chemical shifts of C_α_ nuclei with those for the two neighboring amide nitrogens (Bermel* et al.* 2006b; Bertini* et al.* 2011). Similarly, ^15^N direct-detection experiments, *e*.*g*., CAN and CON (Takeuchi* et al.* 2010b), were acquired as an effective supplement (Fig. 2B). The combination of these experiments enables the assignment of the chemical shifts of the backbone atoms, *i*.*e*., N, Cα, C’. This strategy is also applicable to proline-rich proteins, as amide protons were not required during the assignment. For the case of signal overlap, 3D CANCA (Takeuchi* et al.* 2010a) spectra (Fig. 2C, 2D) were acquired to correlate a given C_αi_ with neighboring N^*i*^ and N^*i* + 1^, which in turn connect to the *i* – 1^th^, *i*^th^ and *i* + 1^th^ C_α_ nuclei. Therefore, the chemical shift connectivity can be straightforwardly established by navigating between C_α_–C_α_ planes in a “stairway” along the nitrogen dimension. These experiments have gained increasing popularity in the sequence-specific assignment of biomolecules, *e*.*g*., proteins, DNAs/RNAs and sugars.

## CHEMICAL SHIFT PERTURBATIONS

**Figure 3:**
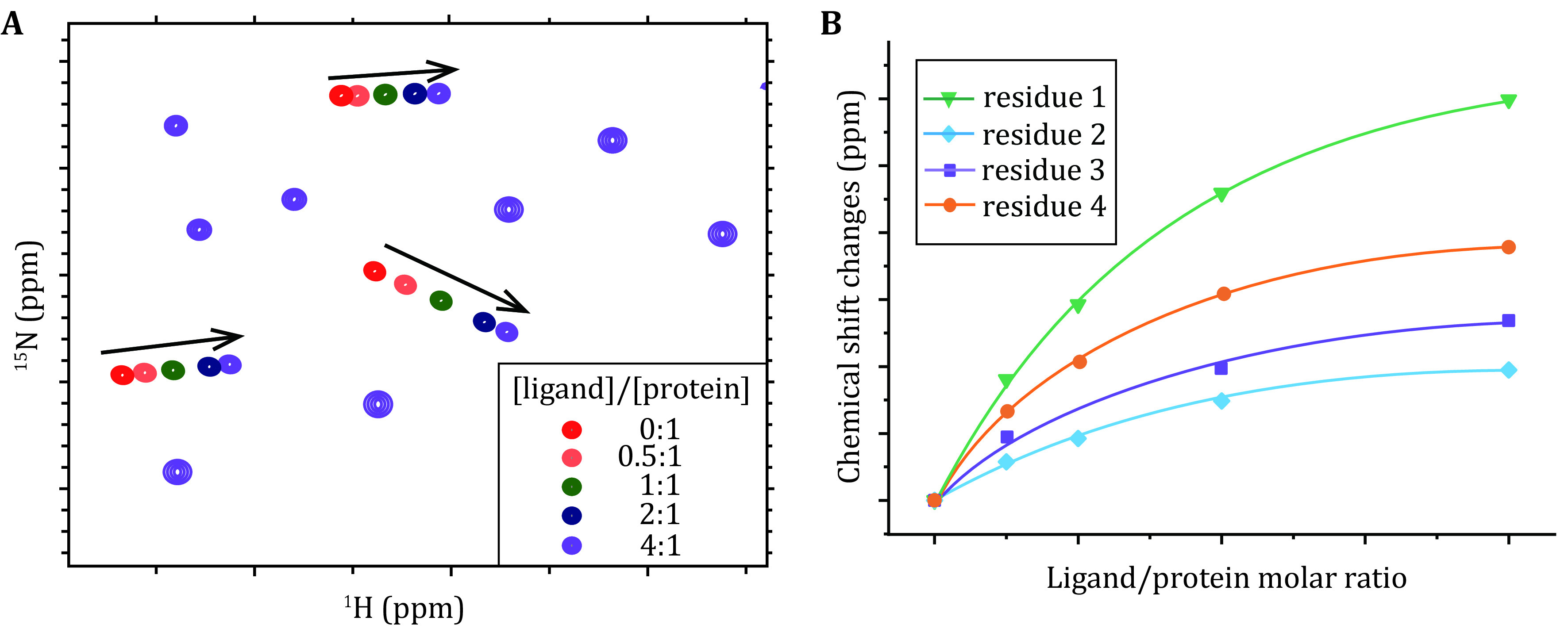
The ligand binding site and affinity are determined from NMR chemical shift perturbations. **A** Illustration of ^1^H-^15^N HSQC spectra obtained for the ^15^N-labeled protein upon titration of a ligand. **B** Best fitting of the dose-dependent chemical shift perturbations to retrieve the dissociation constant

The residue-by-residue CSPs map the ligand-binding topology and affinities for weak protein–protein and protein–RNA interactions. The CSPs were retrieved from the ^1^H-^15^N HSQC spectra and sometimes ^1^H-^13^C HSQC/HMQC spectra for a protein with LLPS tendency upon the titration of the nonlabeled binding partner:

(1) The ^15^N-labeled protein sample is concentrated to approximately 50 μmol/L;

(2) Set the experiment temperature, usually in the range of 5–37 °C. In general, a low temperature is desirable for protein stability and LLPS;

(3) The NMR sample is then tuned into the ^1^H/^13^C/^15^N channel, shimmed, and locked;

(4) Calibrate the 90° pulse width of ^1^H, and ^13^C/^15^N if necessary;

(5) Calibrate the central frequency of the water signal;

(6) Acquire a series of HSQC spectra upon the titration of its binding partner (Fig. 3A).

### Mapping of the binding topology

The HSQC spectra were processed using NMRPipe and analyzed using Sparky.

(1) Load spectral data and parameters in NMRPipe, modify the acquisition mode if necessary;

(2) Run the NMRPipe script to generate a fid file, read this fid file and adjust the 0^th^ and 1^st^ order phase of the ^1^H dimension, while the phases of the indirect dimension were usually set to 0, 0 or –90, 180 if a half-dwell time is applied;

(3) Fourier transform the data along the two dimensions using the NMRPipe script, and then convert to ucsf format;

(4) Pick peaks in Sparky and export the chemical shifts to Origin;

(5) The chemical shift changes, defined as Eq. 1, were calculated statistically residue-by-residue; the residues that demonstrated CSPs of more than two standard deviations from the mean value were considered as direct interacting residues;

(6) These residues were then mapped onto the surface of the NMR/X-ray/CryoEM structure of the protein to determine the binding site.

1 $ \Delta {\delta }_{obs}=\sqrt{1/2[\Delta {\delta }_{\mathrm{H}}^{2}+{\alpha }^{2}{\Delta \delta }_{\mathrm{N}}^{2}]} $

where $ \Delta {\delta }_{\mathrm{H}} $ and $ \Delta {\delta }_{\mathrm{N}} $ denote the chemical shift changes along the ^1^H and ^15^N dimensions, respectively, and α is a constant approximately equal to 0.2.

### Affinity determination

Residues with significant CSPs, *e*.*g*., at least twofold standard deviations above the mean value, were considered for the affinity calculation. Assuming a 1:1 binding mode, the observed CSPs are quantified as follows:

2 $ \Delta {\delta }_{obs}=\frac{\Delta {\delta }_{\mathrm{m}\mathrm{a}\mathrm{x}}\left\{\left({P}_{t}+{L}_{t}+{K}_{\mathrm{d}}\right)-{\left[{\left({P}_{t}+{L}_{t}+{K}_{\mathrm{d}}\right)}^{2}-4{P}_{t}{L}_{t}\right]}^{\tfrac{1}{2}}\right\}}{2{P}_{t}} $

where *P*_*t*_ and *L*_*t*_ denote the concentrations of the protein and ligand, respectively, and *K*_d_ is the dissociation constant as a shared parameter for all residues during the best fitting of the dose-dependent CSPs (Fig. 3B). $ \Delta {\delta }_{\mathrm{m}\mathrm{a}\mathrm{x}} $ represents the maximum of the CSP for each specific residue.

## PARAMAGNETIC RELAXATION ENHANCEMENT

**Figure 4:**
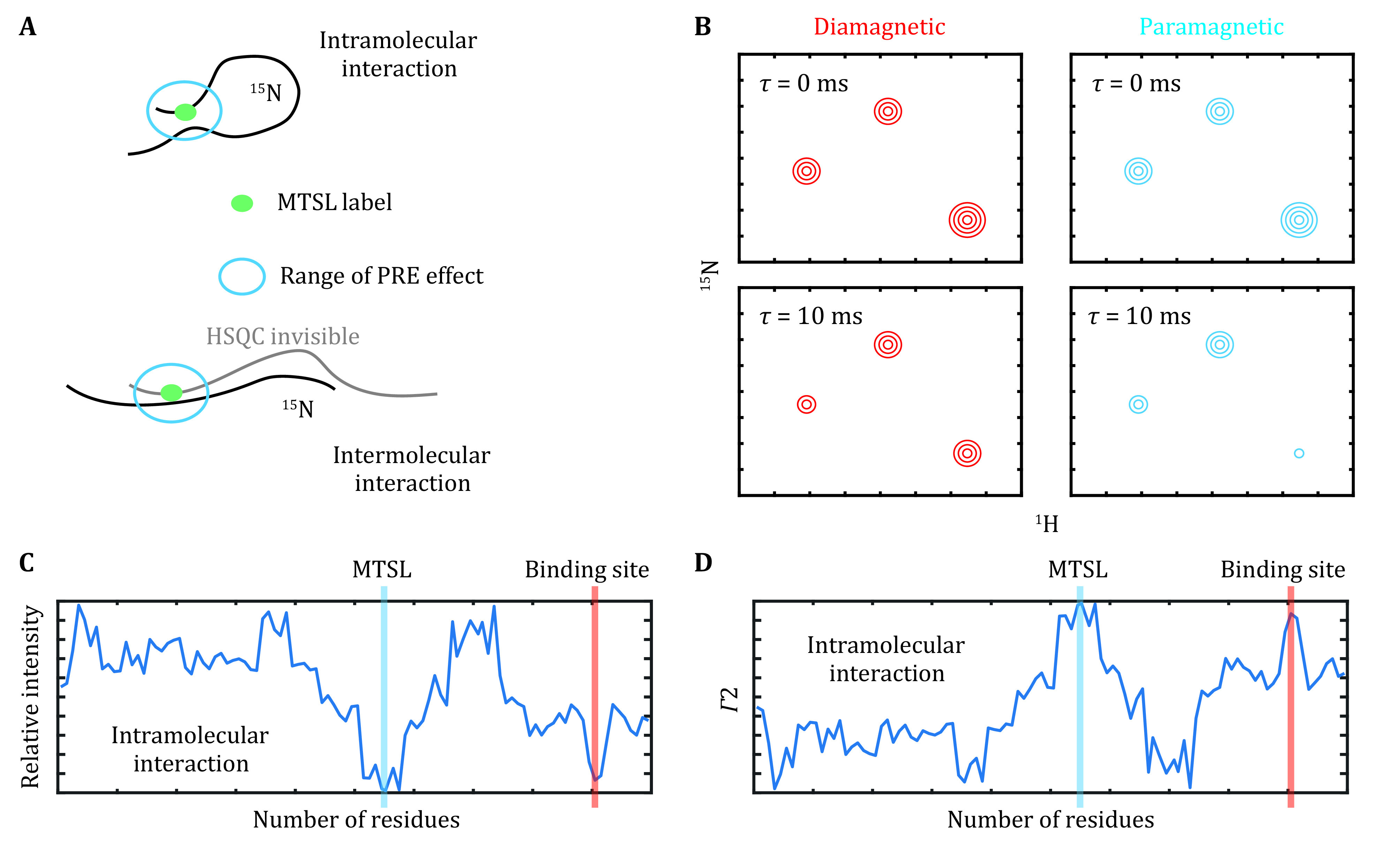
Intramolecular or intermolecular interactions probed by NMR paramagnetic relaxation enhancement. **A** The paramagnetic and isotope labeling scheme for the measurement of paramagnetic relaxation enhancement. **B** Illustration of signal intensities in the paramagnetic or diamagnetic conditions. **C**, **D** The residue-by-residue paramagnetic relaxation enhancement effects determined in a semiquantitative or quantitative way

Paramagnetic relaxation enhancement (PRE) provides distal restraints between the nuclei of interest to the paramagnetic center and is particularly useful for weak protein–protein and protein–RNA interactions (Antoniou and Fung 2008). A paramagnetic labeled protein, *e*.*g*., MTSL covalently linked to a cysteine residue, was first prepared (Fig. 4A). We recommend the acquisition of a ^1^H-^15^N HSQC spectrum for this sample to confirm that MTSL is properly ligated, as evidenced by the disappearance of signals for the residues proximal to this cysteine residue. There are two ways to measure PRE effects as described below.

### Semiquantitative PRE

The normal ^1^H-^15^N HSQC for the ^15^N and MTSL-labeled protein was acquired. This sample was then treated with vitamin C as previously described in the NMR sample preparation section to cleave MTSL from the protein. HSQC spectra with the same parameter settings were then acquired for this reduced sample. The intensity ratio between the paramagnetic and diamagnetic states was depicted in a residue-by-residue manner (Fig. 4B). This approach provides a rough estimation of the distance between the lone-paired electron of MSTL and the residues within a distance of approximately 25 Å.

### Quantitative PRE

The HSQC pulse sequence was modified to measure the ^1^H transverse relaxation rate *R*_2_ (Clore and Iwahara 2009; Iwahara* et al.* 2007). The HSQC-type spectra were then acquired at various relaxation delays. The relaxation rate R2 was best fitted to an exponential decay equation,

3 $ {I}_{t}={I}_{0}{e}^{-{R}_{2}t} $

where *I*_*t*_ and *I*_0_ represent the intensity measured at relaxation T2 delays of *t* and 0 s, respectively. The time *t* was usually optimized to reduce the signal intensities by approximately 30% to 50%.

Accordingly, the paramagnetic (*R*_2, para_) and diamagnetic (*R*_2, dia_) relaxation rates were determined for the MTSL-labeled and vitamin C-reduced samples, respectively (Fig. 4C). The PRE effect, *Γ*_2_, is defined as below,

4 $ {\varGamma }_{2}={R}_{2,\mathrm{p}\mathrm{a}\mathrm{r}\mathrm{a}}-{R}_{2,\mathrm{d}\mathrm{i}\mathrm{a}} $

The residue-by-residue *Γ*_2_ values are proportional to $ {r}_{\mathrm{e}\mathrm{i}}^{-6} $, where *r*_ei_ denotes the distance between the lone-paired electron of the paramagnetic label and the atom of interest (Fig. 4D). The structural model was, thus, optimized to best fit the experimental *Γ*_2_ values.

### Intermolecular PRE

To measure the intermolecular PREs, the protein of interest was ^15^N labeled and mixed with its binding partner, which was MTSL labeled but without isotope labeling. The remaining procedures were the same as those described in the sections "Semiquantitative PRE" or "Quantitative PRE" (Murthy and Fawzi 2020; Zhang* et al.* 2020).

## Conflict of interest

Hanyu Zhang, Weiwei Fan, Gilbert Nshogoza, Yaqian Liu, Jia Gao, Jihui Wu, Yunyu Shi, Xiaoming Tu, Jiahai Zhang and Ke Ruan declare that they have no conflict of interest.
